# *Scn3b* knockout mice exhibit abnormal ventricular electrophysiological properties

**DOI:** 10.1016/j.pbiomolbio.2009.01.005

**Published:** 2008-10

**Authors:** Parvez Hakim, Iman S. Gurung, Thomas H. Pedersen, Rosemary Thresher, Nicola Brice, Jason Lawrence, Andrew A. Grace, Christopher L.-H. Huang

**Affiliations:** a Physiological Laboratory, University of Cambridge, Downing Street, Cambridge CB2 3EG, United Kingdom; b Institute of Physiology and Biophysics, University of Aarhus, DK-8000 C, Denmark; c Takeda Cambridge Limited, Cambridge Science Park, Cambridge CB4 0PA, United Kingdom; d Section of Cardiovascular Biology, Department of Biochemistry, University of Cambridge, Tennis Court Road, Cambridge CB2 1QW, United Kingdom

**Keywords:** Sodium channel, *Scn3b*, Ventricular tachycardia, Brugada syndrome

## Abstract

We report for the first time abnormalities in cardiac ventricular electrophysiology in a genetically modified murine model lacking the *Scn3b* gene (*Scn3b*^−/−^). *Scn3b*^−/−^ mice were created by homologous recombination in embryonic stem (ES) cells. RT-PCR analysis confirmed that *Scn3b* mRNA was expressed in the ventricles of wild-type (WT) hearts but was absent in the *Scn3b*^−/−^ hearts. These hearts also showed increased expression levels of *Scn1b* mRNA in both ventricles and *Scn5a* mRNA in the right ventricles compared to findings in WT hearts. *Scn1b* and *Scn5a* mRNA was expressed at higher levels in the left than in the right ventricles of both *Scn3b*^−/−^ and WT hearts. Bipolar electrogram and monophasic action potential recordings from the ventricles of Langendorff-perfused *Scn3b*^−/−^ hearts demonstrated significantly shorter ventricular effective refractory periods (VERPs), larger ratios of electrogram duration obtained at the shortest and longest S_1_–S_2_ intervals, and ventricular tachycardias (VTs) induced by programmed electrical stimulation. Such arrhythmogenesis took the form of either monomorphic or polymorphic VT. Despite shorter action potential durations (APDs) in both the endocardium and epicardium, *Scn3b*^−/−^ hearts showed ΔAPD_90_ values that remained similar to those shown in WT hearts. The whole-cell patch-clamp technique applied to ventricular myocytes isolated from *Scn3b*^−/−^ hearts demonstrated reduced peak Na^+^ current densities and inactivation curves that were shifted in the negative direction, relative to those shown in WT myocytes. Together, these findings associate the lack of the *Scn3b* gene with arrhythmic tendencies in intact perfused hearts and electrophysiological features similar to those in *Scn5a*^+/−^ hearts.

## 1 Introduction

Voltage-gated sodium (Na^+^) channels, critical for the excitation of cardiac muscle (Keating and Sanguinetti, 2001), are formed of pore-forming *α*-subunits and one or more *β*-subunits (Isom, 2001). Experiments using the two-electrode voltage clamp in *Xenopus* oocytes demonstrated that the *α*-subunits alone can form functional Na^+^ channels (Noda et al., 1986). Thus far nine *α*-subunits, Na_v_1.1–Na_v_1.9 are each encoded by different genes and have been identified and cloned in mammals. Mutations in *SCN5A* which encodes for the cardiac *α*-subunit, Na_v_1.5, can result in arrhythmic conditions, including the Brugada (BrS) and Long QT syndrome (LQTS) (Tan et al., 2003).

However, the electrophysiological roles of *β*-subunits in the function of cardiac Na^+^ channels are less well understood. Six *β*-subunits have been identified and are encoded by 4 different genes; *SCN1B*, *SCN2B*, *SCN3B* and *SCN4B* (Isom et al., 1992, 1995a; Kazen-Gillespie et al., 2000; Morgan et al., 2000; Qin et al., 2003; Yu et al., 2003). These genes, which encode for *β*_1_, *β*_2_, *β*_3_ and *β*_4_ subunits respectively, are expressed in human tissues including the brain, skeletal muscle and the heart (Candenas et al., 2006; Stevens et al., 2001). Of these, the *β*_1_ and *β*_3_ subunits have been demonstrated in the transverse tubules, and *β*_2_ and *β*_4_ subunits in the intercalated disks of murine ventricular myocytes (Maier et al., 2004).

It is possible that the presence of *β*-subunits can affect both the function and expression of the Na^+^ channel. Firstly, studies in heterologous expression systems provided by *Xenopus* oocytes, Chinese hamster ovary (CHO) cells and human embryonic kidney (HEK) cells demonstrated that *β*-subunits modify gating kinetics, including activation, inactivation and the recovery from inactivation of the Na^+^ channel (Chen et al., 2002; Meadows et al., 2002; Morgan et al., 2000; Patton et al., 1994). However, these alterations vary with the particular cell types used to express the Na^+^ channel subunits (Isom et al., 1995b; Morgan et al., 2000; Qu et al., 2001). Secondly, these subunits can also alter the Na^+^ current amplitude (Fahmi et al., 2001; Lopez-Santiago et al., 2007; Watanabe et al., 2008). Thirdly, *β*-subunits contain domains that are structurally homologous to the V-set of the Ig superfamily, which includes cell adhesion molecules (CAMs) (Isom et al., 1995a). This permits cell–cell interaction and adhesion in cells expressing these subunits (Malhotra et al., 2000). Finally, the *β*-subunits have been shown to regulate the expression density of the Na^+^ channel in the plasma membrane. Experiments using CHO and 1610 cell lines have demonstrated a 2- to 4-fold greater Na^+^ channel expression when *α*-subunits were co-expressed with *β*_1_ subunits, than when these were expressed alone (Isom et al., 1995b).

The presence or absence of *β*-subunits has recently been associated with electrophysiological abnormalities in cardiac tissue. Surface electrocardiogram experiments carried out on genetically modified mice, lacking the *Scn1b* gene, have demonstrated a LQT-like phenotype, characterised by a prolonged QT interval (Lopez-Santiago et al., 2007). However, only two clinical reports thus far have associated mutations in the gene *SCN4B* with LQTS (Medeiros-Domingo et al., 2007) and *SCN1B*, encoding both *β*_1_ and *β*_1B_ subunits with BrS (Watanabe et al., 2008). In the present study, we assessed, for the first time, electrophysiological properties in the ventricles of *Scn3b*^−/−^ hearts compared to those shown in WT hearts. Firstly, *Scn3b*^−/−^ mice were created using homologous recombination in embryonic stem (ES) cells. The phenotype of the resulting *Scn3b*^−/−^ mice, including body weight and temperature, appeared similar to that of WT mice. Secondly, ventricles of such mice showed altered expression levels of *Scn3b*, *Scn1b* and *Scn5a* mRNA. Thirdly, studies in Langendorff-perfused *Scn3b*^−/−^ hearts demonstrated arrhythmogenic features. Finally, these corresponded to changes in the biophysical properties of the Na^+^ channel, with features similar to those previously reported in *Scn5a*^+/−^ hearts (Papadatos et al., 2002; Stokoe et al., 2007a).

## 2 Methods

### 2.1 Generation of Scn3b-deficient (−/−) mice

PCR amplification was used to generate homologous arms from the 129SvEv mouse genome, using primers designed to amplify a 5′ arm spanning 2.0 kb of the *Scn3b* locus terminating downstream of the third coding exon splice acceptor (5′armF; tttgcggccgCAGCCTTGTATGAACCCAGGGTCTTTC and 5′armR; tttactagtCACCTCCTGGTGGCCATTTCGATACTC) and a 3′ arm spanning 4.1 kb of the *Scn3b* locus commencing just upstream of the third coding exon splice donor (3′armF; tttggcgcgccGGTAAGCCTGAGGCCTGTAGTCTCTTC and 3′armR; tttggccggccGTGGACTTTAGTCCCATGTCCTCATTG).

The arms were cloned into the plasmid pTK5IBLMNL (Paradigm Therapeutics Ltd, Cambridge, U.K.) using the restriction sites incorporated in the arm primers, such that the 5′ and 3′ arms flank an IRES-fronted *LacZ* reporter gene followed by a *loxP*-flanked PGKNeopA selectable marker. After homologous recombination, 191 bp of the 226 bp third coding exon is replaced by the IRES*LacZ*/PGKneo cassette. Putative targeted embryonic stem (ES) cells were identified by PCR using an external 5′ screening primer (5′scr: CTGACATCTTCTCAGCAGATAACTGAC) in combination with a vector specific primer (DR2; ATCATGGCCCTACCATGCGCTAAACAC). Successfully targeted ES cells were further confirmed by Southern blot of *Afl*II and *Bcl*I digested genomic DNA, using an external probe amplified from a genomic region downstream of the end of the 3′ arm (using the primers 3′prF; AGATGTCCAGCGATACTGTTGAGGCAG and 3′prR; TGTGAGTCTTATTGGAGGTACAGTGTG), indicating that the integration had occurred legitimately.

Correctly targeted 129 SvEv ES cells, containing the *Scn3b* knockout (KO) allele were injected into host blastocysts as previously described (Bradley et al., 1984), generating male chimeras which were subsequently mated with 129 SvEv females. Pups from these crosses were screened with the original target screening PCR (using 5′scr and vector specific primer, DR2), to identify heterozygote animals and in further generations by a multiplex PCR designed to amplify a 204 bp region specific to the WT allele and a 334 bp region specific to the *Scn3b* KO allele. This allowed differentiation of each of the three possible genotypes (using primers hetF, GTCGTCTGCAGTGGAATGGGAGCAAAG; hetR, TGAAGAGACTACAGGCCTCAGGCTTAC; and Asc306, AATGGCCGCTTTTCTGGATTCATCGAC). All genotypes were observed at the expected Mendelian ratios. Routinely homozygous matings were established to produce the experimental cohorts and 129SvEv stock used as the WT controls. Male and female offspring of WT and *Scn3b*^−/−^ mice were randomly selected for use in all experiments. Mice obtained were kept in cages at a room temperature of 21 ± 1 °C in an animal facility with 12-hour light/dark cycles. The mice had free access to sterile rodent chow and water.

A series of tests were used to assess baseline metabolic and behavioural parameters and were performed on cohorts of WT and *Scn3b*^−/−^ mice. Each cohort typically consisted of 5–8 mice of either sex at the age of 3–4 months. The metabolic tests included measuring the body weight and temperature. Each mouse was weighed monthly for up to 3 months and body temperature was measured using a rectal probe (World Precision Instruments, Florida, USA). The behavioural tests measured visual tracking, locomotor activity, touch sensitivity and anxiety. Firstly, visual tracking was assessed using an optokinetic drum apparatus (Paradigm Therapeutics, Cambridge, U.K.). Secondly, locomotor activity was measured as the distance travelled, in one hour, using the LABORAS system (Metris, Hoofddorp, The Netherlands). Finally, the percentage time spent in the open and closed arms of an elevated plus-maze apparatus was used to assess aversion to open spaces in the mice (Lister, 1987). All procedures conformed to the UK Animals (Scientific Procedures) Act 1986.

Randomly selected male and female mice (age 5–8 months) were killed by cervical dislocation in all RT-PCR and *ex vivo* experiments (Schedule 1: UK Animals (Scientific Procedures) Act 1986).

### 2.2 Analysis of sodium channel α- and β-subunit transcripts

To quantify changes in the mRNA expression levels of sodium *α*- and *β*-subunits in hearts obtained from WT and *Scn3b*^−/−^ mice, RT-PCR experiments were performed on an ABI 7500 Fast cycler (Applied Biosystems, Warrington, U.K.). Total RNA was isolated from left atria (LA), right atria (RA), left ventricle (LV) and right ventricle (RV) of WT and *Scn3b*^−/−^ mice (*n* = 3 each) using a Qiagen RNAeasy kit. Excised tissues were stored in RNAlater^®^ (Ambion, Warrington, U.K.) to maintain the integrity of the RNA before isolation. The total RNA was reverse transcribed into cDNA using random hexamer primers and a SuperScript III kit (Invitrogen, Paisley, U.K.). Oligos for *Scn3b, Scn5a,* and *Scn1b* were FAM/TAMRA labelled (Applied Biosystems). All experiments were performed in triplicate.

The number of the copies of mRNA was calculated from its respective threshold cycle (C_T_) using a standard curve. Each value was normalized for the expression value of the housekeeper gene, glyceraldehyde-3-phosphate dehydrogenase (GAPDH) and expressed as a percentage of GAPDH expression (i.e. 2^ΔCT^ × 100).

### 2.3 Preparation of Langendorff-perfused hearts for electrophysiological recordings

The experiments used Langendorff-perfused murine hearts as previously described (Balasubramaniam et al., 2003). Hearts were rapidly excised whilst minimizing contact with the atria and ventricles, then submerged in ice-cold bicarbonate-buffered Krebs–Henseleit solution containing (mM): 119 NaCl, 25 NaHCO_3_, 4.0 KCl, 1.2 KH_2_PO_4_, 1.0 MgCl_2_, 1.8 CaCl_2_, 10 glucose and 2.0 sodium-pyruvate (pH 7.4) and bubbled with 95% O_2_/5% CO_2_ gas mixture (British Oxygen Company, Manchester, U.K.). Under the ice-cold buffer, excess tissues surrounding the heart were removed, leaving a 2–3 mm section of the aorta. This was cannulated and sealed, using a micro-aneurysm clip (Harvard Apparatus, Edenbridge, U.K.) to a 21-gauge tailor-made cannula. The latter was pre-filled with ice-cold buffer solution using a 1 ml syringe. The preparation was transferred and attached to a Langendorff system, and then retrogradely perfused, using the bicarbonate-buffered Krebs–Henseleit solution described above, warmed to 37 °C via a water jacket and circulator (Techne model C-85 A, Cambridge, U.K.). The warmed perfusate was initially passed through a 200 μm and 5 μm filter membrane (Millipore, Watford, U.K.), before being introduced into the aorta at a constant flow of 2–2.5 ml min^−1^ using a peristaltic pump (Watson–Marlow Bredel model 505S, Falmouth, Cornwall, U.K.). The cannulated hearts were perfused for 5 min before further testing. Viable, healthy hearts then regained a homogenous pink colouration and spontaneous rhythmic contraction. Hearts that did not demonstrate these features upon perfusion were instantly discarded.

### 2.4 Bipolar electrogram recording

In all experiments analysing the ventricles of the isolated, perfused murine heart, paired platinum stimulating electrodes (1 mm interpole spacing) were positioned over the epicardial surface of the right ventricle. Ventricular activity was examined by recording from the epicardial surface of the left ventricle using a silver chloride (2 mm tip diameter) recording electrode (Linton Instruments, Harvard Apparatus, U.K.), which was manually positioned. The electrical signals recorded from these hearts resulted in bipolar electrogram (BEG) recordings. The paired platinum stimulating electrodes paced the epicardial surface of the right ventricle and the stimulation used a 2 ms square-wave stimuli at three times excitation threshold (Grass-Telefactor, U.K., Slough, U.K.). These signals were amplified and high-pass filtered for recordings of murine heart (30–1 kHz) using a Gould 2400S amplifier (Gould-Nicolet Technologies, Ilford, Essex, U.K.) and then digitised using an analogue to digital converter (CED 1401plus MKII, Cambridge Electronic Design, Cambridge, U.K.) at a sampling frequency of 5 kHz. All digitised data was then captured and analysed using Spike 2 software (Cambridge Electronic Design).

### 2.5 Monophasic action potential recording and regular pacing

In addition to ventricular BEGs, monophasic action potentials (MAPs) of the ventricles were also recorded using an established contact-electrode technique (Franz, 1999; Killeen et al., 2007; Knollmann et al., 2001). The stimulating electrodes were positioned on the basal surface of the right ventricular epicardium and used to deliver electrical stimulation, as described above. Each isolated perfused heart underwent a period of regular pacing using 2 ms square-wave stimuli, delivered at three times the excitation threshold, at a basic cycle length (BCL) of 125 ms, for up to 20 min to record both epicardial and endocardial MAPs. Epicardial MAPs were recorded from the basal surface of the left ventricular epicardium using the silver chloride recording electrode. Endocardial MAPs were also obtained, from the left ventricular endocardium using a custom-made endocardial MAP electrode, constructed from two Teflon-coated (0.25 mm diameter) silver wires (99.99%) (Advent Research Materials Ltd. Oxford, U.K.), that were twisted together and galvanically chlorided. This electrode was inserted through a small access window carefully created in the interventricular septum (Casimiro et al., 2001). The tip of the electrode was rotated and positioned against the left ventricular free wall, maintaining stable contact pressure. Signals obtained from the recording electrode were amplified and low-pass filtered between 0.1 Hz and 1.0 kHz using the Gould 2400S amplifier (Gould-Nicolet Technologies) and digitised using the CED 1401plus MKII analogue to digital converter (Cambridge Electronic Design) as described above. Both epicardial and endocardial MAP waveforms were analysed using Spike2 software (Cambridge Electronic Design).

MAPs that were used for analysis, satisfied previously established waveform characteristics including a stable baseline, a rapid upstroke phase with consistent amplitude, a smooth contoured repolarization, a stable duration and 100% repolarization achieved by a full return to the baseline (Stokoe et al., 2007a). These MAP waveforms were used to obtain action potential durations (APDs) at 30% (APD_30_), 50% (APD_50_), 70% (APD_70_) and 90% (APD_90_) repolarization. Transmural gradients of repolarization (ΔAPD_90_) were calculated as endocardial APD_90_ value minus the epicardial APD_90_ value (Killeen et al., 2007; Thomas et al., 2007).

### 2.6 Programmed electrical stimulation

Programmed electrical stimulation (PES) was used to assess ventricular arrhythmogenesis in each heart preparation and recorded as BEGs and MAPs (Balasubramaniam et al., 2003; Saumarez and Grace, 2000). The PES protocol first paced the heart for 25 s at the BCL. This was followed by cycles each consisting of an eight stimuli (S_1_) drive train at a CL of 125 ms followed by a ninth extra-stimulus (S_2_). The first S_1_–S_2_ interval (between the eighth S_1_ and S_2_ stimuli) equalled the pacing interval. Successive cycles progressively reduced the S_1_–S_2_ interval by 1 ms until the S_2_ stimulus could no longer evoke a ventricular deflection, at which point, the whole heart preparation reached the ventricular effective refractory period (VERP). VERP was hence defined as the longest S_1_–S_2_ interval that could not elicit a ventricular deflection.

From the ventricular BEGs obtained, conduction curves were constructed, using paced electrogram fractionation analysis (PEFA), a procedure applied in clinical practice (Head et al., 2005; Saumarez and Grace, 2000). These curves plotted the response latencies of each S_2_ extra-stimulus, defined as the time difference between the extra-stimulus and the peak and troughs of the resulting BEG, against the corresponding S_1_–S_2_ interval. The electrogram duration (EGD) is characterised as the time difference between the first and last BEG peak and trough. The EGD ratios from corresponding conduction curves were calculated by normalizing the EGD of the BEG obtained from the shortest S_1_–S_2_ interval with the EGD of the longest S_1_–S_2_ interval (Stokoe et al., 2007a). The initial conduction latency was defined as the time difference from the initial S_2_ stimulus applied at the S_1_–S_2_ interval of 125 ms and the initial deflection of the resulting electrogram response.

### 2.7 Ventricular myocyte isolation

A solution consisting of (in mM): 120 NaCl, 5.4 KCl, 5 MgSO_4_, 5.5 sodium-pyruvate, 10 glucose, 20 taurine and 10 HEPES was used as the basic isolation buffer. This was used to make the following solutions. Firstly, an NTA buffer consisted of the isolation buffer and 5 mM nitrilotriacetic acid (NTA). The pH of this buffer was adjusted to 6.95. Secondly, a digestion buffer, which contained the isolation buffer to which 0.25 mM CaCl_2_, 1 mg ml^−1^ collagenase type 2 (Worthington, NJ, U.S.A.) and 1 mg ml^−1^ hyaluronidase (Sigma–Aldrich, Poole, U.K.) was added. Thirdly, a stop buffer was created, which contained the isolation buffer and 2 mg ml^−1^ bovine serum albumin (Sigma–Aldrich). Fourthly, the isolation buffer to which 0.6 mM CaCl_2_ was added, was created as the wash buffer. Finally, a storage buffer, similar to the wash buffer but with 1.2 mM CaCl_2_, was made. All the buffers created, except for the NTA buffer, had their pH adjusted to 7.4.

Hearts of adult mice were rapidly excised and submerged in ice-cold isolation buffer, prior to whole-heart isolation. The hearts were then perfused for 2 min with NTA buffer in a retrograde fashion using a variable speed peristaltic pump at a flow rate of 3.5 ml ms^−1^ (Autoclude EV045, Essex, U.K.). Before perfusion, the buffer was warmed to 37 °C with a water jacket and circulator (Techne model RB-5 A, Cambridge, U.K.). Following this, the heart was then perfused in the same fashion with the digestion buffer for 12–15 min. Using watchmaker's forceps, ventricular tissue was taken from the whole heart and placed in the stop buffer. After transferring into a 15 ml test tube, gentle trituration was used to isolate single myocytes from the harvested cardiac tissue. After 5 min for the cells to settle, the supernatant was then gently removed. The isolated cells were then washed using wash buffer. Following a further 5 min, the isolated myocytes were resuspended and stored in the storage buffer. After each step after harvesting the cells, isolated myocytes were checked under a light microscope to confirm cell integrity. Live myocytes appeared as smooth and rod-shaped cells.

### 2.8 I_Na_ measurements

The biophysical experiments studied the activation, inactivation and recovery from inactivation in the Na^+^ channels of WT and *Scn3b*^−/−^ myocytes. These experiments were carried out at a physiological temperature of 37 °C, using the whole-cell configuration, with an Axopatch 700B amplifier (Molecular Devices, Berkshire, U.K.). Borosilicate glass pipettes (0.86 mm outer diameter; Harvard Apparatus, Kent, U.K) with a resistance of 1.5–2.5 MΩ were used. These pipettes were filled with (in mM): 70.26 CsCl, 24.74 Cs-aspartate, 10 HEPES, 1 Na_2_ATP, 4 MgATP, 1.37 MgCl_2_, and 10 Cs-BAPTA, adjusted to pH 7.2 using CsOH. Cells were loaded into a chamber which was mounted on the stage of a Nikon TE2000 Eclipse microscope (Nikon, Kingston, U.K.). These cells were initially perfused with an extracellular bath solution, which contained (in mM): 145 NaCl, 5 KCl, 10 glucose, 10 HEPES, 1 MgCl_2_ and 1 CaCl_2_, adjusted to pH 7.4 using NaOH. To achieve optimum voltage control, Na^+^ currents were recorded in a low [Na^+^] solution. This solution contained (in mM): 10 NaCl, 140 CsCl, 10 glucose, 10 HEPES, 1 MgCl_2_ and 1 CaCl_2_, adjusted to pH 7.4 using CsOH. The currents obtained were filtered at a 10 kHz high frequency cut off and digitised at 100 kHz using pClamp 10 software (Molecular Devices). In each experiment, the series resistance was compensated by at least 70% and each protocol used a holding potential of −100 mV.

Firstly, the voltage dependence of activation was studied by applying depolarizing pulses of 20 ms duration. These pulses were applied over series of voltage steps from −100 mV, in 10 mV increments, to 70 mV. The resulting currents were normalized against the cell capacitance (*C*_m_) and plotted against time. The peak current densities (pA/pF) were calculated by dividing the peak currents amplitude by *C*_m_. The current–voltage relationship was obtained by plotting the values of the peak current density against each corresponding membrane voltage (*V*_m_). The values of peak Na^+^ conductance (*g*_Na_) values were determined from the equation

$$g_{\text{Na}}=I_{\text{Na}}/\left(V_{\text{m}}-E_{\text{Na}}\right)\text{,}$$

where *I*_Na_ was the peak current density and the *E*_Na_ was the [Na^+^] reversal potential. The *E*_Na_ was assumed to be 42.6 mV. This was based on the Nernst equation;

$$E_{\text{Na}}=\left(RT/zF\right)\ln\left({\left[{\text{Na}}^{+}\right]}_{\text{extracellular}}/{\left[{\text{Na}}^{+}\right]}_{\text{intracellular}}\right)\text{,}$$

where *R* is the universal gas constant, *T* is the absolute temperature at 310 K, *z* is the valence of the ionic species at 1, *F* is Faraday's constant, [Na^+^]_extracellular_ = 10 mM and [Na^+^]_intracellular_ = 2 mM. The *g*_Na_ values calculated from each myocyte were normalized against the corresponding peak *g*_Na_ value. When plotted against the test-pulse voltage, the data assumed a sigmoid function, increasing with membrane potential.

Secondly, the voltage dependence of inactivation was examined using pre-pulses of 500 ms duration. These pre-pulses were applied over a voltage range from −120 mV to −20 mV in 10 mV increments. Each pre-pulse was followed by a test pulse to −20 mV at a duration of 10 ms. The peak currents obtained were normalized against the peak current recorded at the pre-pulse of −120 mV. The normalized currents were then plotted against the pre-pulse voltage. The data assumed a sigmoid function, decreasing with membrane potential. Both inactivation and activation data were fitted with a Boltzmann function:

$$y={\left[1+\exp\left\{(V_{\text{m}}-V_{1/2})/k\right\}\right]}^{-1}$$

yielding the half-maximal voltage (*V*_1/2_) and slope factor (*k*) values. The *y* values corresponded to the normalized current values.

Finally, to determine the time course of recovery from inactivation, a protocol consisting of 18 cycles was employed. Each cycle consisted of a 1 s pre-pulse to −20 mV, used to achieve inactivation of the Na^+^ channels, followed by a return to the voltage of −100 mV to allow recovery of the channels. With each cycle, the duration of recovery increased from 1 to 250 ms. A 50 ms test pulse to −20 mV determined the current from the recovered channels. The currents recorded were normalized against the current obtained from each pre-pulse applied. A bi-exponential function was fitted to the recovery from inactivation data,

$$y=y_{0}+A_{\text{f}}\left\{1-\exp\left(-t/\tau_{\text{f}}\right)\right\}+A_{\text{s}}\left\{1-\exp\left(-t/\tau_{\text{s}}\right)\right\}\text{,}$$

where *A*_f_ and *A*_s_ are the amplitude of the fast and slow inactivating components, and *τ*_f_ and *τ*_s_ are the time constants of the fast and slow components, respectively. The *y* values correspond to the normalized current values. Data was analysed using Signal 2.16 (Cambridge Electronic Design) and all curve fits were performed using OriginPro 8 (OriginLab Corporation, MA, USA).

### 2.9 Data analysis and statistical procedures

For experiments in mRNA expression levels, the single-factor analysis of variance for independent samples (ANOVA) test was used to compare the difference between WT and *Scn3b*^−/−^ tissue samples (Microsoft EXCEL Analysis ToolPak) to show significant changes in expression of the mRNA.

Whole-heart BEG and MAP data were initially imported into Microsoft EXCEL. The ANOVA test was used to compare WT and *Scn3b*^−/−^ data sets (Microsoft EXCEL Analysis ToolPak). Calculated values of *P* < 0.05 were considered significant. Results are shown as means ± S.E.M values.

An *F*-test was used firstly to confirm the normal distribution of the data acquired from the Na^+^ current recordings (Microsoft EXCEL Analysis ToolPak) made in WT and *Scn3b*^−/−^ myocytes. Depending on the type of variance, the appropriate form of the Student *t* test was applied to analyze the differences between two groups of data.

## 3 Results

### 3.1 Gene targeting of Scn3b and generation of knockout mice

**Fig. 1:**
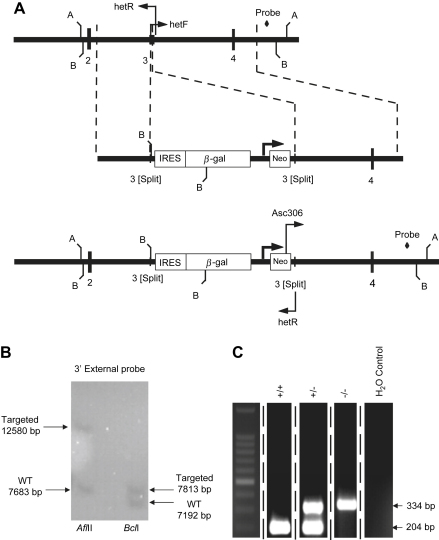
Targeted deletion of the *Scn3b* gene. (*A*) Schematic representation of the *Scn3b* allele that was targeted for deletion. The top line shows a partial restriction map of the *Scn3b* locus, indicating the restriction sites for *Afl*II (*A*) and *Bcl*I (*B*). The centre line represents the targeted vector and the predicted targeted allele after homologous recombination shown in the bottom line. The location of the PCR primers and the probe used for screening the correctly targeted embryonic stem (ES) cells and the mice are shown. IRES; internal ribosome entry site, β-gal; beta-galactosidase gene and Neo; neomycin resistance gene. (*B*) Southern blot analysis of a successfully targeted ES cell. Digestion with *Afl*II yielded a 7.7 kb endogenous band and a 12.6 kb bp targeted band as predicted. Digestion with *Bcl*I resulted in a 7.2 kb endogenous band and the predicted 7.8 kb bp targeted band. (*C*) PCR analysis identified band sizes of 204 bp in the wild-type (+/+) allele and 334 bp in homozygote knockout (−/−) allele. Both bands were observed in the heterozygote (+/−) allele. A water control was used to assess the specificity of the primers used. All 5 lanes shown were obtained from the same gel.

To disrupt the *β*_3_ coding region, 191 bp of the 226 bp third exon of the *β*_3_ sequence was replaced by the IRES*LacZ*/pGKneo cassette (Fig. 1*A*). The 5′ arm of the plasmid used to generate the mutant contained a 2.0 kb fragment of the *Scn3b* locus terminating downstream of the third coding exon splice acceptor, and a 3′ arm which spanned 4.1 kb of the *Scn3b* locus commencing just upstream of the third coding exon splice donor. Successfully targeted ES cells were screened by Southern blot using an external 3′ probe (amplified from mouse genomic DNA using 3′prF; AGATGTCCAGCGATACTGTTGAGGCAG and 3′prR; TGTGAGTCTTATTGGAGGTACAGTGTG). Digestion with *Afl*II yielded a 7.7 kb endogenous band and a 12.6 kb bp targeted band as predicted. Similar experiments using *Bcl*I resulted in a 7.2 kb endogenous band and the predicted 7.8 kb bp targeted band (Fig. 1*B*). Homozygous mutant (*Scn3b*^−/−^) offspring were identified using PCR screening (Fig. 1*C*). The primers used (hetF, GTCGTCTGCAGTGGAATGGGAGCAAAG; hetR, TGAAGAGACTACAGGCCTCAGGCTTAC; and Asc306, AATGGCCGCTTTTCTGGATTCATCGAC) resulted in the generation of a 204 bp band from the wild-type (WT) (+/+) allele, and a 334 bp band from the *Scn3b*^−/−^ (−/−) allele. Both bands were present in the heterozygote (+/−) allele. A water control was used to confirm the specificity of the PCR primers.

To assess the phenotype of the *Scn3b*^−/−^ mice, both metabolic and behavioural tests were used. The body weight and temperature of these mice were similar to those obtained from WT mice (Table 1). Additionally, vision, locomotor activity and anxiety tests showed similar results in both WT and *Scn3b*^−/−^ mice (Table 1).

### 3.2 mRNA expression levels of Na^+^ channel subunits in murine ventricles

The mRNA expression studies demonstrated that *Scn3b* mRNA was not present and an increase in *Scn1b* mRNA in both the left and right ventricles of *Scn3b*^−/−^ hearts. An increase in the *Scn5a* mRNA was observed only in the right ventricles of *Scn3b*^−/−^ hearts. In all cases, the mRNA expression in the left ventricles was consistently higher. Reverse-transcriptase PCR (RT-PCR) was used to investigate mRNA expression levels of sodium (Na^+^) channel *β*-subunits *Scn3b*, *Scn1b*, and the cardiac Na^+^ channel *α*-subunit, *Scn5a*, in tissue obtained from the right and left ventricles of mouse wild-type (WT) (RV+/+ and LV+/+; *n* = 3) and *Scn3b*^−/−^ hearts (RV−/− and LV−/−; *n* = 3). The number of the copies of mRNA was calculated from its respective threshold cycle (C_T_) using a standard curve. Each value was normalized to the corresponding expression of the housekeeping gene, glyceraldehyde-3-phosphate dehydrogenase (GAPDH).

**Fig. 2:**
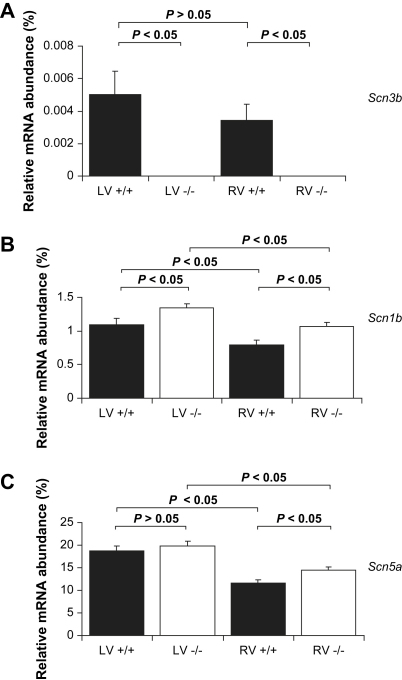
RT-PCR analysis of mRNA expression. The relative abundance of mRNA (normalized and calculated as % of GAPDH expression) of encoding Na^+^ channel subunit transcripts. The histograms show data from the left and right ventricles (LV and RV respectively) of wild-type (black) and *Scn3b*^−/−^ (white) mice. (*A*) mRNA transcripts for *Scn3b* was present in ventricles of WT (left; LV +/+, right; RV +/+, *n* = 3) and not detectable in ventricles of *Scn3b*^−/−^ mice (left; LV −/−, right; LV −/−, *n* = 3). (*B*) The expression levels of *Scn1b* mRNA were significantly higher in both the right and left ventricles of *Scn3b*^−/−^ hearts than shown in WT hearts (*P* < 0.05). (*C*) The *Scn5a* transcripts were significantly higher in the right but not in the left ventricles of *Scn3b*^−/−^ hearts. mRNA expression levels of *Scn3b*, *Scn1b* and *Scn5a* were consistently higher in the left than in the right ventricles of both WT and *Scn3b*^−/−^ hearts (*n* = 3 each, *P* < 0.05) with the exception of *Scn3b* mRNA expression levels in *Scn3b*^−/−^ hearts.

This made comparison between samples possible by correcting for any variation between the quantity of mRNA isolated from the WT and transgenic mouse hearts. Transcript expression was expressed as relative abundances of mRNA expression, calculated as a % of the GAPDH expression (i.e. 2^ΔCT^ × 100). Firstly, the mRNA encoding the *β*_3_ subunits, *Scn3b*, was expressed at statistically similar levels in both the left and right ventricles of WT hearts (Fig. 2*A*, black column, *n* = 3, *P* < 0.05). In contrast, both the ventricles of *Scn3b*^−/−^ hearts (*n* = 3), as expected, did not show any detectable expression of *Scn3b* mRNA.

Secondly, both the left and right ventricles of the *Scn3b*^−/−^ hearts (Fig. 2*B,* white column) showed an increase in the expression levels of *Scn1b* mRNA, relative to corresponding values in WT hearts (*P* < 0.05). Finally, Fig. 2*C* confirms the expression of the mRNA encoding for the α-subunit of the Na^+^ channel, *Scn5a*, in both ventricles of WT (black columns) and *Scn3b*^−/−^ (white columns) hearts. The left ventricles of WT and *Scn3b*^−/−^ hearts demonstrated similar levels of *Scn5a* mRNA expression (*P* > 0.05). However, the right ventricles of the *Scn3b*^−/−^ hearts demonstrated a small but significant increase in the expression of *Scn5a* mRNA, than that shown in WT hearts (*P* < 0.05). Thus, mRNA expression levels of *Scn1b* and *Scn5a* were always higher in the left than in the right ventricles of WT and *Scn3b*^−/−^ hearts (*n* = 3 each, *P* < 0.05).

### 3.3 Analysis of BEG recordings during PES

The mRNA expression changes were correlated with a ventricular arrhythmogenic phenotype in *Scn3b*^−/−^ hearts. This was demonstrated in electrophysiological experiments that assessed ventricular arrhythmogenicity in isolated, Langendorff-perfused WT and *Scn3b*^−/−^ murine hearts. This was achieved by obtaining bipolar electrogram (BEG) (first series; 6 WT hearts and 7 *Scn3b*^−/−^ hearts) and monophasic action potential (MAP) (second series; 14 WT hearts and 17 *Scn3b*^−/−^ hearts, third series; 4 WT hearts and 4 *Scn3b*^−/−^ hearts) recordings from the left ventricles. BEGs and MAPs were obtained once the preparations had regained a steady state after 10 min following mounting. BEG recordings were obtained during programmed electrical stimulation (PES). This procedure has been used in clinical situations to assess arrhythmogenicity and applied to murine hearts as described on previous occasions (Balasubramaniam et al., 2003; Saumarez and Grace, 2000). Hearts were initially paced at a basic cycle length (BCL) of 125 ms for 25 s. Subsequent cycles used a drive train each consisting of eight stimuli (S_1_) at the same BCL, followed by a premature extra-stimulus (S_2_). The initial S_2_ stimulus was imposed at an S_1_–S_2_ interval of 125 ms. With each cycle, this interval was reduced by 1 ms, until either the ventricular effective refractory period (VERP) was reached, at which point the premature S_2_ did not trigger a response or resulted in ventricular tachycardia (VT).

**Fig. 3:**
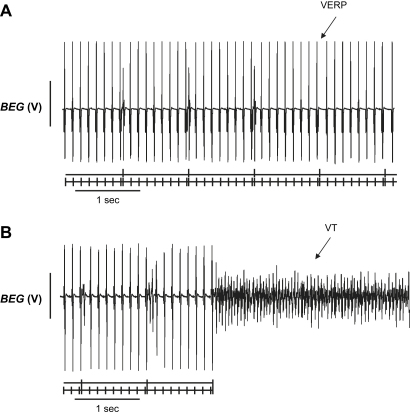
BEG recordings of programmed electrical stimulation (PES) from WT and *Scn3b*^−/−^ hearts. (*A*) PES of an isolated, WT Langendorff-perfused heart. Ventricular effective refractory period (VERP) was obtained from all WT hearts studied (*n* = 6). (*B*) BEG recording showing PES of an isolated, *Scn3b*^−/−^ Langendorff-perfused heart. PES induced ventricular tachycardia (VT) in 5 out of 7 *Scn3b*^−/−^ heart preparations.

Fig. 3 shows examples of BEG traces obtained during PES. Each trace demonstrates results from the final cycles that corresponded to the shortest S_1_–S_2_ intervals. The single vertical marks below each recording indicate the successive S_1_ stimuli and the double vertical marks, the S_2_ extra-stimuli. Furthermore, each trace contains stimulus artefacts that occurred at regular CL, followed by their resulting electrogram. In the case of WT hearts (Fig. 3*A*), shortening of the S_1_–S_2_ interval led to an S_2_ extra-stimulus that did not elicit an electrogram. This S_1_–S_2_ interval corresponded to the VERP. In contrast, under such circumstances, a significant proportion of *Scn3b*^−/−^ hearts showed ventricular arrhythmias (Fig. 3*B*).

**Fig. 4:**
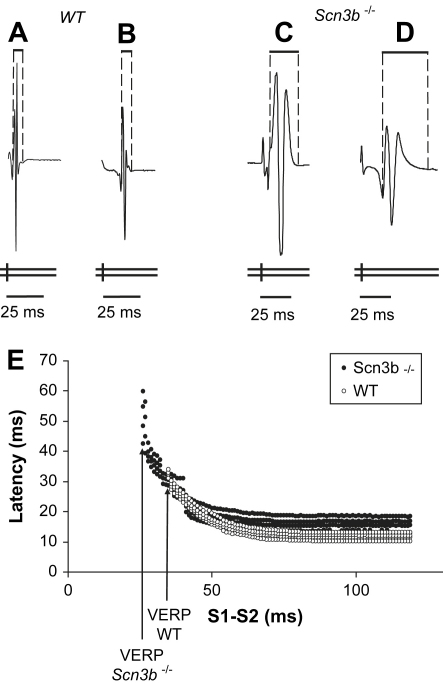
Electrogram waveforms and conduction curves from programmed electrical stimulation (PES) and bipolar electrogram (BEG) recordings. The electrograms recorded at the longest S_1_–S_2_ interval (basic cycle length of 125 ms) in WT hearts (*A*) were significantly shorter in duration than in *Scn3b*^−/−^ hearts (*C*). Similarly, electrograms obtained prior to the heart becoming refractory or arrhythmic were consistently shorter in duration in WT hearts (*B*) than those recorded in *Scn3b*^−/−^ hearts (*D*). (*E*) Conduction curves were created by the application of paced electrogram fractionation analysis (PEFA) to PES data obtained from WT (white circles) and *Scn3b*^−/−^ hearts (black circles). The vertical axis represents latency (ms) and the horizontal axis, the S_1_–S_2_ interval (ms). The representative conduction curve demonstrates a greater increase in electrogram duration at the shortest S_1_–S_2_ interval and shorter VERP in *Scn3b*^−/−^ hearts, than recorded in WT hearts.

Conduction curves were then derived from the BEG recordings obtained during PES, using paced electrogram fractionation analysis (PEFA), a procedure that has been used to assess clinical risk of ventricular arrhythmogenicity on earlier occasions (Saumarez and Grace, 2000). It has also been previously applied to assess arrhythmogenicity in *Kcne1*^−/−^ (Balasubramaniam et al., 2003), *Scn5a*^+/ΔKPQ^ (Head et al., 2005; Stokoe et al., 2007b) and *Scn5a*^+/−^ (Stokoe et al., 2007a) murine hearts. Fig. 4*A*–*D* illustrates S_2_-induced BEG waveforms at a faster time base than shown in Fig. 3 in order to demonstrate differences in electrogram duration (EGD). This was defined as the time interval between the first and last deflection of each electrogram waveform. These waveforms were obtained during PES of WT (Fig. 4*A*, *B*) and *Scn3b*^−/−^ (Fig. 4*C*, *D*) hearts. The waveforms in Fig. 4*A*, *C* were induced by the S_2_ stimuli at the longest S_1_–S_2_ intervals that were identical to the BCL, employed at the beginning of the procedure. The duration of these waveforms were defined as the initial EGD. The waveforms in Fig. 4*B*, *D* were induced by the S_2_ stimuli at the shortest S_1_–S_2_ intervals that could elicit an electrogram response, at the end of the procedure. The duration of these waveforms were defined as the final EGD. The EGD ratio was calculated as the final EGD divided by the initial EGD. The initial conduction latency was calculated as the time difference from the initial S_2_ extra-stimulus applied at the S_1_–S_2_ interval of 125 ms and the resulting BEG.

Fig. 4*E* shows examples of the conduction curves in WT and *Scn3b*^−/−^ hearts. Each of the curves plots the latencies of each peak and trough of each deflection contained in the electrogram from the S_2_ stimulus against the corresponding S_1_–S_2_ interval. Such curves demonstrate increases in the degree of spread of each electrogram and conduction latency as S_1_–S_2_ interval shortens. However, these changes are more marked in *Scn3b*^−/−^ hearts. A greater increase in the EGD was observed in *Scn3b*^−/−^ hearts with shortening of the S_1_–S_2_ interval, (*D*, filled circles) compared to the EGD shown in WT hearts (*B*, clear circles). Fig. 4*E* also demonstrates the significantly shorter VERP observed in *Scn3b*^−/−^ hearts than in WT hearts. Tables 2 and 3 summarise the VERP, initial and final EGD values, EGD ratios and conduction latencies obtained from the PEFA analysis of WT and *Scn3b*^−/−^ hearts. It was possible to determine a VERP in only 2 out 7 *Scn3b*^−/−^ hearts, with the remaining 5 hearts demonstrating PES-induced VT (Table 3). Nevertheless, the mean S_1_–S_2_ interval at which VERP and arrhythmogenicity occurred in *Scn3b*^−/−^ hearts was 24.7 ± 1.6 ms (*n* = 7 hearts), which was significantly shorter than the VERP of the WT hearts studied (31.2 ± 1.5 ms, *n* = 6, *P* < 0.05). *Scn3b*^−/−^ hearts showed significantly larger initial and final EGD values (17.1 ± 3.8 ms and 34.6 ± 6.6 ms respectively, *n* = 7; Table 3) compared to those of WT hearts (2.3 ± 0.4 ms and 2.8 ± 0.4 ms, *n* = 6, *P* < 0.05; Table 2). Furthermore, *Scn3b*^−/−^ hearts demonstrated a larger mean EGD ratio than that shown in WT hearts (2.1 ± 0.2 ms, *n* = 7 vs. 1.3 ± 0.1 ms *n* = 6, *P* < 0.05). WT and *Scn3b*^−/−^ hearts both showed similar conduction latencies (8.0 ± 0.4 ms vs. 8.3 ± 0.2 ms *P* > 0.05; Tables 2 and 3).

### 3.4 Analysis of MAP recordings during PES

**Fig. 5:**
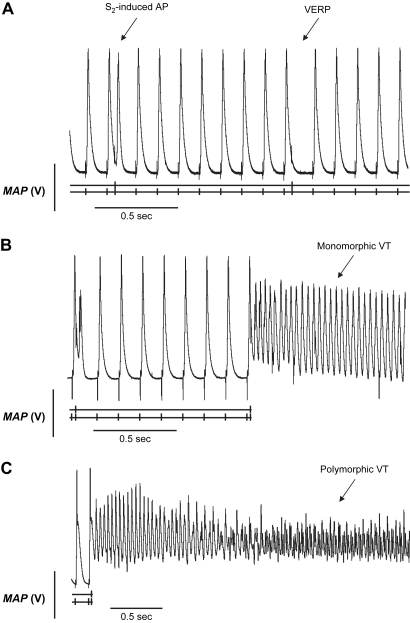
Monophasic action potential (MAP) recordings of PES from WT and *Scn3b*^−/−^ hearts. PES of isolated Langendorff-perfused WT (*A*) and *Scn3b*^−/−^ (*B*, *C*) hearts. S_2_-induced action potential (AP) and VERP was observed in all WT hearts studied (*n* = 14). (*B*) PES-induced episodes of VT occurred in 10 out of 17 *Scn3b*^−/−^ hearts, with five such preparations demonstrating a monomorphic VT. (*C*) An episode of PES-induced VT in a *Scn3b*^−/−^ heart preparation began as a monomorphic VT and deteriorated into polymorphic VT.

Analysis of MAP recordings permitted a closer differentiation of hearts with arrhythmogenic and non-arrhythmogenic phenotypes as well as of monomorphic and polymorphic VT. MAP waveforms were compared during PES in a second series of isolated, perfused WT and *Scn3b*^−/−^ hearts. Each heart was subjected to an application of a single procedure of PES. This made it possible to determine when VT occurred, whether it was monomorphic or polymorphic. Fig. 5 illustrates MAP recordings obtained from WT (*A*) and *Scn3b*^−/−^ (*B*, *C*) hearts during the final cycles of a PES procedure that corresponded to the shortest S_1_–S_2_ intervals. In each panel, the single vertical mark below each recording indicates the S_1_ stimuli and the double vertical mark the S_2_ extra-stimulus. The traces also show the corresponding stimulus artefacts that occurred at a regular CL, closely followed by their elicited MAP. MAPs in response to S_1_ stimuli were consistent in waveform in both WT and *Scn3b*^−/−^ hearts, with each AP demonstrating a stable baseline, rapid upstroke and smooth repolarisation phase (Fabritz et al., 2003; Guo et al., 1999; Killeen et al., 2007). Neither demonstrated spontaneous events such as early or delayed afterdepolarizations that were reported in hypokalaemic and *Scn5a*^+/ΔKPQ^ murine hearts (Killeen et al., 2007; Thomas et al., 2008).

Fig. 5*A* shows a typical MAP recording obtained during the final cycles of the PES procedure in a WT heart. The first S_2_ extra-stimulus that is displayed, gave rise to an action potential. However, the heart was refractory in response to the second S_2_ extra-stimulus, thereby permitting the determination of the VERP. In contrast, *Scn3b*^−/−^ hearts showed episodes of either monomorphic (Fig. 5*B*) or polymorphic VT (Fig. 5*C*) following an S_2_ extra-stimulus. Monomorphic VT showed successive ventricular deflections that were of similar waveform and amplitude. Polymorphic VT demonstrated varying intervals between each successive deflection with irregular amplitudes and waveforms. Table 4 summarises the properties of the MAP waveforms during PES in WT (*n* = 14) and *Scn3b*^−/−^ hearts (*n* = 17). It lists the VERP shown in non-arrhythmogenic hearts. Where the heart was arrhythmogenic, it lists the S_1_–S_2_ interval value, which would be less than the VERP. The WT hearts had a mean VERP of 39.3 ± 1.6 ms (*n* = 14). PES did not induce VT in any of the WT hearts. In contrast to WT hearts, only 7 out of 17 *Scn3b*^−/−^ hearts were non-arrhythmogenic. These hearts had a mean VERP of 43.3 ± 4.4 ms (*n* = 7). This was not significantly different from the corresponding mean VERP, recorded in WT hearts (*n* = 14, *P* > 0.05). The remaining *Scn3b*^−/−^ hearts were arrhythmogenic in response to a mean S_1_–S_2_ interval of <29.3 ± 2.4 ms (*n* = 10). This was significantly shorter than the VERP of the WT (*n* = 14) and non-arrhythmogenic *Scn3b*^−/−^ hearts (*n* = 7; *P* < 0.05). The mean duration of the VT was 98.85 ± 39.8 s (Fig. 5*B, C*). In 5 out of the 10 arrhythmogenic hearts, the hearts showed monomorphic VT (Fig. 5*B*). The mean duration of episodes of monomorphic VT was 100.6 ± 46.4 s (Table 5). In 4 hearts out of 10 arrhythmogenic hearts, the hearts demonstrated polymorphic VT. The mean duration of the polymorphic VT was 27.3 ± 13.8 s. Finally, PES-induced VT in the remaining heart began as a monomorphic VT and deteriorated into polymorphic VT (Fig. 5*C*). This persisted until spontaneous termination of the episode.

### 3.5 Endocardial and epicardial MAP recordings in WT and Scn3b^−/−^ hearts

Comparison of endocardial and epicardial recordings suggested that *Scn3b*^−/−^ hearts showed a shorter endocardial and epicardial APD_90_ which nevertheless demonstrated a normal transmural gradient of repolarization, ΔAPD_90_. Previous studies have demonstrated that arrhythmogenic properties were associated with abnormal differences in epicardial and endocardial APDs (Killeen et al., 2007; Stokoe et al., 2007b). The present investigation made similar experiments, which recorded MAPs at the BCL from the left ventricular epicardium and endocardium of a third series of hearts. Measurements were obtained from five separate 20 s periods during regular pacing. For each period taken, the recording electrode was repositioned to obtain independent readings from different regions of the ventricle. This was to ensure the values obtained were comparable. Action potential duration (APD) at 30% (APD_30_), 50% (APD_50_), 70% (APD_70_) and 90% (APD_90_) repolarization values were obtained from the MAPs.

**Fig. 6:**
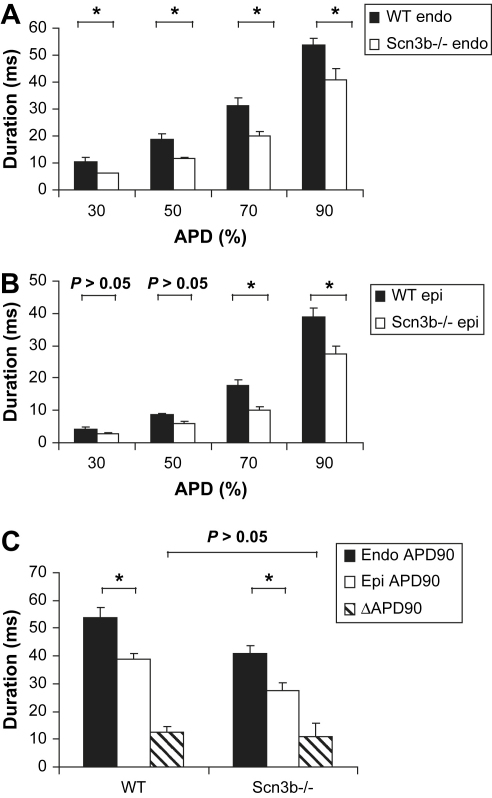
Action potential durations (APDs) in WT and *Scn3b*^−/−^ hearts. Comparison of endocardial (endo) (*A*) and epicardial (epi) (*B*) APD values, measured at 30%, 50%, 70% and 90% repolarization for WT (*n* = 4) and *Scn3b*^−/−^ hearts (*n* = 4) (black and white columns respectively). All endocardial APD recordings, epicardial APD_70_ and APD_90_ were significantly shorter. (*C*) Endocardial and epicardial APD measured at 90% repolarization (APD_90_) values (black and white columns respectively), alongside ΔAPD_90_ values for WT and *Scn3b*^−/−^ hearts, which showed no significant difference (striped column; *P* > 0.05). *P < 0.05.

Tables 6 and 7 summarise the APD values obtained from the epicardium and endocardium. Endocardial APD values in *Scn3b*^−/−^ hearts were significantly shorter than their corresponding WT endocardial values. Thus, the endocardial APD_30_, APD_50_, APD_70_ and APD_90_ recorded in WT hearts (*n* = 4) were 10.5 ± 1.4, 18.8 ± 2.1, 31.2 ± 2.9 and 53.6 ± 2.7 ms respectively (Fig. 6*A*). The same measurements made in *Scn3b*^−/−^ hearts demonstrated APD values of 6.2 ± 0.1 ms, 12.3 ± 1.2 ms, 20.2 ± 1.6 ms and 41.0 ± 3.8 ms (*n* = 4, *P* < 0.05). Furthermore, *Scn3b*^−/−^ epicardial APD_70_ and APD_90_ values were also significantly shorter than their respective WT APD_70_ and APD_90_ values. Thus, the epicardial APD_70_ and APD_90_ values obtained from WT hearts were 17.7 ± 1.6 ms and 38.7 ± 2.8 ms respectively (Fig. 6*B*). Equivalent measurements obtained from *Scn3b*^−/−^ hearts were 10.2 ± 0.8 ms and 27.5 ± 2.3 ms respectively (*n* = 4, *P* < 0.05). Epicardial APD_30_ and APD_50_ values were both similar in WT and *Scn3b*^−/−^ hearts (*P* > 0.05). The transmural gradient of repolarization (ΔAPD_90_) was quantified as the difference between the values of the endocardial APD_90_ and epicardial APD_90_ (Killeen et al., 2007). The *Scn3b*^−/−^ and WT hearts showed similar positive ΔAPD_90_ (14.0 ± 3.4 ms vs. 15.4 ± 4.4 ms, *P* > 0.05) (Fig. 6*C*). This is despite a decrease in epicardial and endocardial values observed in *Scn3b*^−/−^ hearts.

### 3.6 Na^+^ current recordings in isolated ventricular myocytes

The electrophysiological changes in the *Scn3b*^−/−^ whole heart was correlated with a reduction in the peak Na^+^ current and a shift in the inactivation curve in the negative direction. The activation properties and recovery from inactivation remained similar in WT and *Scn3b*^−/−^ myocytes. The effects of the expression of *β*_3_ subunits on Na^+^ currents were assessed using the whole-cell patch-clamp technique on cardiac myocytes isolated from WT and *Scn3b*^−/−^ hearts. In all experiments, the series resistance was compensated by at least 70% and each protocol used a holding voltage of −100 mV.

**Fig. 7:**
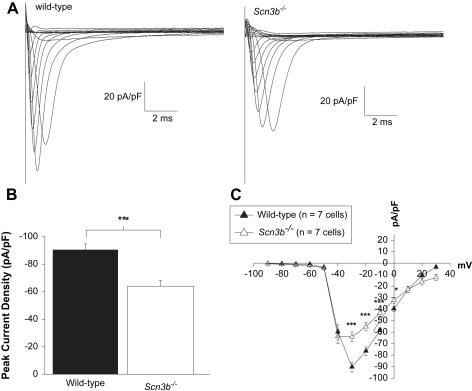
Peak Na^+^ current densities in WT and *Scn3b*^−/−^ myocytes. Representative Na^+^ current traces recorded from WT (*A*, left panel) and *Scn3b*^−/−^ (*A*, right panel) myocytes. (*B*) The peak Na^+^ current densities were significantly smaller in *Scn3b*^−/−^ myocytes (white column, *n* = 7) compared to those shown in WT myocytes (black column, *n* = 7). (*C*) The current–voltage relationship demonstrated a smaller Na^+^ current in *Scn3b*^−/−^ myocytes (white triangles, *n* = 7) between the voltage steps of −30 and 0 mV compared to those shown in WT myocytes (black triangles, *n* = 7). *P < 0.05, ***P < 0.001. All recordings were obtained at 37 °C.

Firstly, Na^+^ current densities and voltage dependence of activation were assessed using a pulse protocol which applied depolarizing pulses of 20 ms duration, from a holding potential of −100 mV, to test potentials between −90 and 70 mV in 10 mV increments. Fig. 7*A* compares families of representative current traces obtained from WT (left panel) and *Scn3b*^−/−^ (right panel) myocytes in response to voltage steps, between the test potentials of −100 to 30 mV. In all the WT and *Scn3b*^−/−^ myocytes examined, the greatest magnitude of peak Na^+^ current density was observed at voltage steps to either −30 or −40 mV. However, the maximum value of the peak Na^+^ current density was significantly smaller in the *Scn3b*^−/−^ myocytes, compared to that shown in WT myocytes (Fig. 7*A*). Thus, Fig. 7*B* demonstrates that the maximum value of the peak current density at −30 mV was significantly reduced by 30% in *Scn3b*^−/−^ myocytes when compared to that shown in WT myocytes (90.4 ± 4.3 pA/pF, *n* = 7 vs. 63.3 ± 4.8 pA/pF, *n* = 7 each, *P* < 0.001; Table 8). Furthermore, Fig. 7*C* plots the peak Na^+^ current densities obtained from WT and *Scn3b*^−/−^ myocytes against the corresponding test potentials. The peak Na^+^ current densities in *Scn3b*^−/−^ myocytes (Fig. 7*C*, white triangles) at −30 (*P* < 0.001), −20 (*P* < 0.001), −10 (*P* < 0.001) and 0 mV (*P* < 0.05) were significantly smaller than the corresponding densities recorded in WT myocytes (black triangles, *n* = 7 each).

**Fig. 8:**
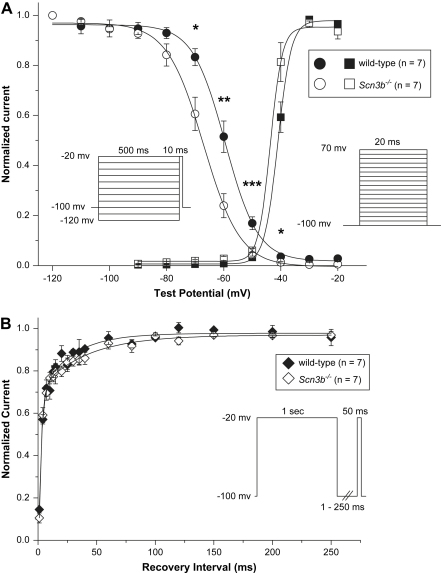
Steady-state activation, inactivation and recovery from inactivation properties in WT and *Scn3b*^−/−^ hearts. (*A*) Steady-state voltage dependence of activation (square symbols) and inactivation (circle symbols) in WT (black symbols) and *Scn3b*^−/−^ (white symbols) myocytes. The solid lines represent the Boltzmann fits. The normalized inactivation currents were significantly smaller in *Scn3b*^−/−^ myocytes (*n* = 7) between the voltage steps of −70 and −40 mV compared to those shown in WT myocytes (*n* = 7). The pulse protocols used to study the voltage dependence of activation and inactivation are shown in the inset. (*B*) Recovery from inactivation in WT (black diamonds) and *Scn3b*^−/−^ (white diamonds) myocytes. The solid lines represent double-exponential fits. The derived biophysical properties are summarised in Table 8. *P < 0.05, **P < 0.01, ***P < 0.001. All recordings were obtained at 37 °C.

Secondly, to examine changes in the biophysics of the Na^+^ channel activation and inactivation properties were assessed in WT and *Scn3b*^−/−^ myocytes. The activation function of Na^+^ channels (Fig. 8*A*, squares) in WT (black) and *Scn3b*^−/−^ (white) myocytes was obtained by plotting the peak Na^+^ conductance (*g*_Na_) (peak *g*_Na_) values against *V*_m_. Peak *g*_Na_ values were calculated by the following formula;

$$g_{\text{Na}}=I_{\text{Na}}/\left(V_{\text{m}}-E_{\text{Na}}\right)\text{,}$$

where *I*_Na_ was the peak current density, *V*_m_ was membrane voltage and the *E*_Na_ was the reversal potential of Na^+^ ions. This was calculated from the intracellular and extracellular Na^+^ concentration as an assumed value of 42.6 mV. The peak *g*_Na_ values obtained were normalized to the corresponding maximum peak *g*_Na_ values.

Thirdly, the voltage dependence of inactivation was assessed using pre-pulses of 500 ms duration. These pre-pulses were applied over a voltage range from −120 to −20 mV in 10 mV increments. A test pulse to −20 mV of duration of 10 ms immediately followed this. The resulting currents were normalized against the Na^+^ current recorded at the pre-pulse at −120 mV. The inactivation function (Fig. 8*A*, circles) in WT (black) and *Scn3b*^−/−^ (white) myocytes was obtained by plotting the normalized currents against *V*_m_. The continuous lines display the Boltzmann functions fitted to the activation and inactivation data. The data was used to obtain the half-maximal voltages (*V*_1/2_) and slope factor (*k*) values of both activation and inactivation in WT and *Scn3b*^−/−^ myocytes (Table 8). The activation function of the Na^+^ channel in WT and *Scn3b*^−/−^ myocytes remained similar, despite a smaller Na^+^ current recorded in *Scn3b*^−/−^ myocytes. Thus, the *V*_1/2_ and *k* values of WT (−41.0 ± 0.2 mV and 2.4 ± 0.3 mV) were similar to those in *Scn3b*^−/−^ hearts (−43.6 ± 0.4 mV and 2.1 ± 0.2 mV, *n* = 7 each, *P* > 0.05). However, the inactivation curve of the Na^+^ channels in *Scn3b*^−/−^ myocytes was shifted in the negative direction relative to the corresponding curve in WT myocytes. Thus, the *V*_1/2_ in the voltage dependence of inactivation of WT and *Scn3b*^−/−^ myocytes was −59.5 ± 0.4 mV and −67.0 ± 0.4 mV respectively (*n* = 7 each, *P* < 0.01).

However, the slope factors of the inactivation curves were similar in WT and *Scn3b*^−/−^ myocytes (WT; 5.7 ± 0.3 mV vs. *Scn3b*^−/−^; 6.4 ± 0.4 mV, *n* = 7 each, *P* > 0.05). At the holding potential of −100 mV, the normalized inactivation current was 0.95 in both WT and *Scn3b*^−/−^ myocytes (Fig. 8*A*). However, at a depolarized potential of −80 mV, the normalized values were 0.93 and 0.84 in WT and *Scn3b*^−/−^ myocytes respectively. This suggests that at −80 mV, an additional 10% of the channels in *Scn3b*^−/−^ myocytes were already inactivated, compared to those in WT myocytes. Furthermore, the percentage of inactivated channels in *Scn3b*^−/−^ myocytes, compared to WT myocytes, increases to 37% at a potential of −70 mV. This was calculated from the normalized current values of 0.83 and 0.61 in WT and *Scn3b*^−/−^ myocytes respectively.

Finally, the time course of recovery from inactivation was examined using a 1 s pre-pulse to −20 mV. This was followed by a return to the holding voltage of −100 mV to allow recovery, at varying durations between 1 and 250 ms. A test pulse at a duration of 50 ms to −20 mV determined the current observed in fractions of recovered channels. The currents recorded were normalized against the current obtained from each of the 1 s pre-pulses applied. To determine the relative proportion of channels that were in the fast (*τ*-fast) and slow (*τ*-slow) phase, a 2-exponential function was fitted to the data (Fig. 8*B*, Table 8). No significant differences were observed in the time constants and fractions of the *τ*-fast (WT; 2.3 ± 0.5 ms and 0.83 ± 0.07, *n* = 7 vs. *Scn3b*^−/−^; 2.1 ± 0.2 ms and 0.97 ± 0.04, *n* = 7, *P* > 0.05) and *τ*-slow (WT; 27.1 ± 7.5 ms and 0.3 ± 0.05, *n* = 7 vs. *Scn3b*^−/−^; 39.5 ± 5.3 ms and 0.27 ± 0.02, *n* = 7, *P* > 0.05) values obtained (Fig. 8*B*).

## 4 Discussion

Sodium (Na^+^) channel function is critical for normal initiation, conduction and propagation of cardiac electrical activity (Grant, 2001). Thus, mutations in the *SCN5A* gene, which encodes for the pore-forming *α*-subunit of the cardiac Na^+^ channel Na_v_1.5, result in clinical arrhythmic conditions that include the Brugada syndrome (BrS) (Brugada and Brugada, 1992; Chen et al., 1998) and long QT syndrome 3 (LQT3) (Wang et al., 1995). These conditions have been reproduced in genetically modified, *Scn5a*^+/−^ and *Scn5a*^+/ΔKPQ^, murine models, respectively (Head et al., 2005; Papadatos et al., 2002). Murine models have also been useful in studying the consequences of alterations in the potassium (K^+^) channel, such as mutations in the *KCNE1* subunit resulting in LQT5 (Balasubramaniam et al., 2003; Vetter et al., 1996) and of acute conditions such as hypokalaemia (Killeen et al., 2007).

There have been fewer studies of *β*- than *α*-subunits of ion channels in murine systems, particularly those associated with the Na^+^ channel. One or more of such *β*-subunits associate with an *α*-subunit to form the complete channel (Catterall, 2000). So far, six types of Na^+^ channel *β*-subunits have been identified and cloned. Of these, the *β*_1_ (Isom et al., 1992), *β*_2_ (Isom et al., 1995a), *β*_3_ (Morgan et al., 2000) and *β*_4_ subunits (Yu et al., 2003) are encoded by *SCN1B*, *SCN2B*, *SCN3B* and *SCN4B* respectively. The remaining *β*_1A_ (Kazen-Gillespie et al., 2000) and *β*_1B_ (Qin et al., 2003) subunits are generated by splice variants of *SCN1B*. mRNA transcripts encoding for these *β*_1_, *β*_2_, *β*_3_ and *β*_4_ subunits are expressed in a wide range of human tissues that include brain, skeletal (Candenas et al., 2006) and cardiac muscle (Isom et al., 1995b; Makita et al., 1994; Morgan et al., 2000; Qin et al., 2003; Yu et al., 2003). The expression of these subunits is thought to be important in normal surface expression (Isom et al., 1995b) and determining the biophysical characteristics of the Na^+^ channel (Chen et al., 2002; Meadows et al., 2002; Morgan et al., 2000; Patton et al., 1994). It has also been suggested that cleaved forms of the *β*-subunits may act as transcription factors for mRNA transcripts encoding for Na^+^ channel subunits. Thus, the cleaved intracellular domain of the *β*_2_ subunit has been shown to increase the expression of Na_v_1.1 *α*-subunits (Kim et al., 2007). Furthermore, the ventricles of *Scn1b*^−/−^ hearts have greater expression levels of *Scn5a* mRNA than do WT hearts (Lopez-Santiago et al., 2007). Modifications in *β*-subunits thus have potential effects on cardiac electrophysiology.

We report, for the first time, that deficiencies in the *Scn3b* gene in a murine system lead to altered biochemical and biophysical properties of the Na^+^ channel, resulting in abnormal ventricular electrophysiology. *Scn3b*^−/−^ mice were first created using homologous recombination in embryonic stem (ES) cells. Such mice showed no significant changes in behavioural and metabolic tests compared to those assessed in WT mice. These findings are in contrast to those shown in *Scn1b*^−/−^ mice, which demonstrate ataxic gait, generalized seizures and growth retardation (Chen et al., 2004). Reverse-transcriptase polymerase chain reaction (RT-PCR) analysis not only confirmed the presence or absence of *Scn3b* mRNA in WT and *Scn3b*^−/−^ hearts respectively, but also that the *Scn3b* mRNA or protein may exert a regulatory role on the expression of *Scn1b* and *Scn5a* mRNA in murine ventricles. Firstly, it demonstrated that *Scn3b* mRNA was expressed in wild-type (WT) with similar expression in both the left and right ventricles. This was in agreement with previous studies that had demonstrated that *β*_3_ subunits were expressed in the ventricular myocytes obtained from WT and *Scn1b*^−/−^ mouse hearts, specifically in the transverse tubules (Lopez-Santiago et al., 2007; Maier et al., 2004). As expected, *Scn3b* mRNA was not expressed in *Scn3b*^−/−^ hearts. Secondly, the *Scn1b* and *Scn5a* mRNA in both WT and *Scn3b*^−/−^ ventricles were expressed at higher levels compared to the expression of *Scn3b* mRNA in WT hearts, confirming a previous report concerning such experiments in WT hearts (Marionneau et al., 2005). Thirdly, *Scn1b* mRNA was expressed at higher levels in *Scn3b*^−/−^ hearts than in WT hearts. These levels of expression were greater in the left than in the right ventricles in both WT and *Scn3b*^−/−^ hearts. Finally, *Scn5a* mRNA was expressed at similar levels in the left ventricles of WT and *Scn3b*^−/−^ hearts. It was expressed at higher levels in the right ventricles of *Scn3b*^−/−^ hearts, compared to levels in the right ventricles of WT hearts.

Electrophysiological studies in the ventricles of the whole intact *Scn3b*^−/−^ hearts also showed arrhythmogenesis and conduction abnormalities. This arrhythmogenesis took the form of either monomorphic or polymorphic ventricular tachycardias (VTs). These electrophysiological findings showed both similarities and contrasts to earlier results obtained from experiments on arrhythmogenic murine *Scn5a*^+/−^, *Scn5a*^+/ΔKPQ^, *Kcne1*^−/−^ and hypokalaemic hearts, as well as human BrS (Table 9(A)). The murine systems had been used to model human BrS, LQT3, LQT5 and hypokalaemia, respectively (Balasubramaniam et al., 2003; Head et al., 2005; Killeen et al., 2007; Papadatos et al., 2002). Firstly, the *Scn3b*^−/−^ hearts showed VT during programmed electrical stimulation (PES) and paced electrogram fractionation analysis (PEFA) of these results demonstrated abnormal conduction curves, in common with the remaining examples in Table 9(A). These curves showed that *Scn3b*^−/−^ hearts had electrogram duration (EGD) ratios that were significantly longer but conduction latencies that were similar at the S_1_–S_2_ interval of 125 ms as those shown in WT hearts. Secondly, the *Scn3b*^−/−^ hearts showed shorter ventricular effective refractory periods (VERPs) than in WT hearts, in common with findings from hypokalaemic hearts and clinical cases of BrS but in contrast to features shown by the remaining murine models. Finally, *Scn3b*^−/−^ hearts had shorter endocardial and epicardial APD_90_ values than did WT hearts. Nevertheless, the difference between these values in *Scn3b*^−/−^ hearts, which offers a measure of the transmural gradient of repolarization in the form of the ΔAPD_90_, was similar to that shown by WT hearts. This is in common with findings in *Scn5a*^+/−^ hearts but in contrast to the remaining examples.

The use of the whole-cell patch-clamp technique demonstrated the loss of function of the cardiac Na^+^ channel in ventricular myocytes isolated from *Scn3b*^−/−^ hearts. These experiments were carried out at the same temperature of 37 °C as the studies used on intact hearts in contrast to previous investigations that had carried out similar single cell experiments at room temperature (Lopez-Santiago et al., 2007; Papadatos et al., 2002). Table 9(B) compares the resulting Na^+^ channel properties in *Scn3b*^−/−^ myocytes with those shown in *Scn5a*^+/−^ and *Scn5a*^+/ΔKPQ^ myocytes and points to a number of important comparisons. Firstly, *Scn3b*^−/−^ myocytes showed significantly smaller peak Na^+^ current densities, in response to depolarizing voltage steps from a −100 mV holding potential, than did WT myocytes. This was in common with previous findings in *Scn5a*^+/−^ myocytes but in contrast to those shown in *Scn5a*^+/ΔKPQ^ myocytes (Table 9(B)). This suggests a reduced expression or proportion of functional Na^+^ channels in the plasma membrane of *Scn3b*^−/−^ ventricles compared to that shown in WT ventricles. The present experiments were performed under conditions of higher ratios of extracellular and intracellular [Na^+^]. This may account for the larger Na^+^ current densities observed in the present study, compared to those observed in previous investigations (Nuyens et al., 2001; Papadatos et al., 2002). Both latter studies used lower extracellular and intracellular [Na^+^] ratios of 2:1 and 2:2 respectively and carried out single cell experiments at room temperature (22–23 °C). Such conditions had resulted in peak Na^+^ current densities of ∼15 pA/pF and 21 ± 5 pA/pF respectively. Secondly, the Na^+^ channel activation properties in *Scn3b*^−/−^ myocytes were similar to those of WT myocytes in common with findings in *Scn5a*^+/−^ and *Scn5a*^+/ΔKPQ^ myocytes. However, the present findings were obtained at a higher temperature of 37 °C than used in the previous studies (Lopez-Santiago et al., 2007; Papadatos et al., 2002). This may account for the similarly highly rapid time courses of activation and small slope factors of steady-state activation observed in both the WT and *Scn3b*^−/−^ myocytes. Thirdly, the position of the inactivation curves in *Scn3b*^−/−^ myocytes was nevertheless shifted in the negative direction relative to that shown in WT myocytes, whereas both *Scn5a*^+/−^ and *Scn5a*^+/ΔKPQ^ myocytes showed normal inactivation curves. At a typical resting membrane potential between −80 and −70 mV (Guo et al., 1999; Remme et al., 2006; Yao et al., 1998), such a shift would result in the inactivation of a further 10 to 37% of the Na^+^ channels in *Scn3b*^−/−^ myocytes, compared to that shown in WT myocytes. Finally, the time course in the recovery from inactivation in *Scn3b*^−/−^ myocytes were similar to that shown in WT myocytes in common with findings in *Scn5a*^+/ΔKPQ^ myocytes.

Together, the altered peak Na^+^ current density and inactivation features shown in *Scn3b*^−/−^ myocytes are compatible with a reduction of the total *in vivo* Na^+^ current. This is compatible with findings in BrS. Thus, although the clinical cases of BrS have shown a wide range of alterations in the activation, inactivation and recovery from inactivation properties, all show reduced Na^+^ currents. The present findings also add a further example of BrS that involves mutations in the Na^+^ channel *β*-subunit. A previous investigation had identified a single mutation in the *SCN1B* gene and two mutations in the splice variant of the same gene in patients with BrS (Watanabe et al., 2008). It used a heterologous expression system provided by Chinese hamster ovary (CHO) cells, to study the consequent changes in the biophysical properties of the Na^+^ channel resulting from the mutations. In agreement with the present example, cells expressing both the WT Na_v_1.5 and such mutant *β*-subunits showed significantly reduced peak Na^+^ currents. In contrast, activation and inactivation properties of the Na^+^ channel shifted in the positive direction in relation to findings in cells expressing WT Na_v_1.5 and WT *β*-subunits. The present findings also complement other investigations in which alterations in the *β*-subunit expression can produce other electrophysiological changes that include a LQT-like phenotype. For example, Lopez-Santiago et al. (2007) associated a deletion of the *Scn1b* gene in a murine system with an increase in peak Na^+^ currents and QT prolongation.

The present findings complement reports of altered Na^+^ channel biophysical properties resulting from *β*_3_ expression in heterologous expression systems by describing the consequences of similar expression *in vivo*. However, the properties of such *in vitro* expression systems may not correspond to features observed *in vivo* and have demonstrated conflicting results despite the transfection of identical sets of genes. Such systems lack functionally important proteins such as ankyrin proteins that form complexes with the Na^+^ channel *in vivo* (Abriel and Kass, 2005). Thus, on the one hand, *Xenopus* oocytes injected with cRNA for Na_v_1.5 and *β*_3_ subunits demonstrated significantly larger peak Na^+^ currents and positive shifts in the inactivation curves than in cells expressing Na_v_1.5 only (Fahmi et al., 2001). In contrast, CHO cells expressing Na_v_1.5 and *β*_3_ subunits resulted in similar peak Na^+^ currents with negative shifts in the activation and inactivation curves compared to cells expressing Na_v_1.5 only (Ko et al., 2005).

In conclusion, we have for the first time associated the lack of the *Scn3b* gene with a ventricular arrhythmogenesis in intact perfused hearts whose features are similar to those shown in *Scn5a*^+/−^ hearts.

## Figures and Tables

**Table 1:** Metabolic and behavioural tests applied to WT and *Scn3b*^−/−^ mice.

Phenotypic test	WT mice	*Scn3b*^−/−^ mice	Result
Body temperature (°C)	37.7 ± 0.1 (*n* = 35)	38.09 ± 0.1 (*n* = 9)	*P* > 0.05
Body weight at 3 months (g)			
Males	26.6 ± 0.7 (*n* = 20)	26.9 ± 1.2 (*n* = 5)	*P* > 0.05
Females	21.4 ± 0.5 (*n* = 23)	21.9 ± 0.8 (*n* = 4)	*P* > 0.05
Visual tracking	Present	Present	
Distance travelled (m)	17.69 ± 1.8 (*n* = 10)	14.28 ± 2.3 (*n* = 9)	*P* > 0.05
Plus Maze			
% time in closed arm	56.1 ± 4.6 (*n* = 10)	52.0 ± 7.1 (*n* = 10)	*P* > 0.05
% time in open arm	2.2 ± 1.0 (*n* = 10)	2.2 ± 0.9 (*n* = 10)	*P* > 0.05

**Table 2:** Ventricular effective refractory period, EGDs and latencies measured in BEG recordings of (“−” = absent; “+” = present).

WT						
Heart	Arrhythmogenic?	VERP (ms)	Initial EGD (ms)	Final EGD (ms)	EGD ratio	Latency (ms)
1	−	27	1.87	3	1.6	7.9
2	−	35	2.2	2.2	1.0	9.6
3	−	28	4.06	4.6	1.1	8.3
4	−	36	1.4	1.8	1.3	8.2
5	−	32	1.3	1.9	1.5	6.7
6	−	29	2.84	3.4	1.2	7.4

Mean ± S.E.M.		31.2 ± 1.5	2.3 ± 0.4	2.8 ± 0.4	1.3 ± 0.1	8.0 ± 0.4

**Table 3:** Ventricular effective refractory period, S_1_–S_2_ interval of PES-induced VT, EGDs and latencies measured in BEG recordings of *Scn3b*^−/−^ hearts.

*Scn3b*^−/−^						
Heart	Arrhythmogenic?	VERP (ms)	Initial EGD (ms)	Final EGD (ms)	EGD ratio	Latency (ms)
1	+	<21	30.5	62.2	2.0	8.3
2	+	<23	16.0	36.2	2.3	8.2
3	+	<33	31.4	55.0	1.8	8.6
4	−	24	12.8	26.4	2.1	7.5
5	−	26	5.6	17.3	3.1	9.6
6	+	<26	11.9	21.8	1.8	8.0
7	+	>20	11.6	23.1	2.0	8.3

Mean ± S.E.M.		24.7 ± 1.6^a^	17.1 ± 3.8^a^	34.6 ± 6.6^a^	2.1 ± 0.2^a^	8.3 ± 0.2^b^

a *P* < 0.05 vs. WT (see Table 2).

b *P* > 0.05 vs. WT (see Table 2) (“−” = absent; “+” = present).

**Table 4:** Ventricular effective refractory periods and S_1_–S_2_ (+) intervals of PES-induced VT in MAP recordings of WT and *Scn3b*^−/−^ hearts.

Heart	WT		Heart	*Scn3b*^−/−^			
	Arrhythmogenic?	VERP (ms)		Arrhythmogenic?	VERP (ms)	Duration of VT (sec)	Type of VT
1	−	46	1	−	40	–	–
2	−	42	2	−	<41	20.715	Poly
3	−	35	3	−	50	–	–
4	−	38	4	−	31	–	–
5	−	32	5	−	<36	21.54	Mono
6	−	36	6	−	<31	376.176	Mono → Poly
7	−	46	7	−	30	–	–
8	−	49	8	+	45	–	–
9	−	36	9	+	<22	150.386	Mono
10	−	30	10	+	<31	1.11	Poly
11	−	35	11	+	64	–	–
12	−	40	12	+	<31	21.24	Poly
13	−	47	13	+	<22	72.5	Mono
14	−	38	14	+	<37	3.1	Mono
			15	+	43	–	–
			16	+	<24	255.62	Mono
			17	+	<18	66.16	Poly

Mean ± S.E.M		39.3 ± 1.6		Arrhythmogenic (+)	<29.3 ± 2.4^a^	98.9 ± 39.8 (*n* = 10)	–
				Non-arrhythmogenic (−)	43.3 ± 4.4^b^		

Poly: Polymorphic VT. Mono: Monomorphic VT.

a *P* < 0.05 vs. WT.

b *P* > 0.05 vs. WT.

**Table 5:** Number of arrhythmogenic *Scn3b*^−/−^ hearts, in MAP recordings, showing PES-induced monomorphic ventricular tachycardia (mVT), polymorphic ventricular tachycardia (pVT) or both (mVT + pVT).

Type of VT	No. of hearts	Mean duration ± S.E.M. (sec)
mVT	5	100.6 ± 46.4
pVT	4	27.3 ± 13.8
mVT + pVT	1	376.2

Total VT	10	98.9 ± 39.8

**Table 6:** Endocardial monophasic APDs of WT and *Scn3b*^−/−^ hearts.

Parameters (ms)	Endocardial	
	Wild-type (*n* = 4)	*Scn3b*^−/−^ (*n* = 4)
APD_30_	10.5 ± 1.4	6.2 ± 0.1^a^
APD_50_	18.8 ± 2.1	12.3 ± 1.3^a^
APD_70_	31.2 ± 2.9	22.0 ± 1.4^a^
APD_90_	53.6 ± 2.7	41.0 ± 3.8^a^

a *P* < 0.05 vs. WT.

**Table 7:** Epicardial monophasic APDs of WT and *Scn3b*^−/−^ hearts.

Parameters (ms)	Epicardial	
	Wild-type (*n* = 4)	*Scn3b*^−/−^ (*n* = 4)
APD_30_	4.1 ± 0.8^b^	2.8 ± 0.4^b^
APD_50_	8.5 ± 0.6^b^	5.8 ± 1.0^b^
APD_70_	17.7 ± 1.6^b^	10.2 ± 0.8a,b
APD_90_	38.7 ± 2.8^b^	27.5 ± 2.3a,b

a *P* < 0.05 vs. WT.

b *P* < 0.05 vs. Endocardial value (in Table 6).

**Table 8:** *I*_Na_ properties in myocytes.

		Wild-type (*n* = 7)	*Scn3b*^−/−^(*n* = 7)
Peak *I*_Na_ density (pA/pF)		−90.4 ± 4.3	−63.3 ± 4.8***

Voltage dependence of activation	*V*_1/2_ (mV)	−41.0 ± 0.2	−43.6 ± 0.4
	*k* (mV)	2.3 ± 0.3	2.1 ± 0.2

Voltage dependence of inactivation	*V*_1/2_ (mV)	−59.5 ± 0.4	−67.0 ± 0.4**
	*k* (mV)	5.7 ± 0.3	6.4 ± 0.4

Recovery from inactivation	*τ*-Fast (ms)	2.3 ± 0.5	2.1 ± 0.2
	*A*_f_	0.83 ± 0.07	0.97 ± 0.04
	*τ*-Slow (ms)	27.1 ± 7.5	39.5 ± 5.3
	*A*_s_	0.3 ± 0.05	0.27 ± 0.02

** *P* < 0.01, *** *P* < 0.001.

**Table 9:** Alterations in electrophysiological features in the hearts of *Scn3b*^−/−^ mice relative to WT hearts, compared with corresponding changes in arrhythmogenic murine models and clinical Brugada Syndrome.

Features	*Scn3b*^−/−^	Arrhythmogenic *Scn5a*^+/−^	Arrhythmogenic *Scn5a*^+/ΔKPQ^	*Kcne1*^−/−^	Hypokalaemic	Clinical BrS	References
(A) Whole heart properties							
PES VT	+	+	+	+	+	+	1–5
Increased EGD	+	+	+	+	*Not assessed*	+	2, 3, 5, 6
Initial conduction latency	No change	No change	Increased^3^	No change	*Not assessed*	*Not assessed*	2, 3, 5, 7
			No change^7^				
VERP	Shorter	No change^2^	No change	No change	Shorter	Short	2, 3, 5, 7–10, 23
		Longer^9^	Longer^3^				
ΔAPD_90_	No change	No Change	Negative	Negative	Negative	Varied	2, 4, 7, 11–13

(B) Single cell properties							
Peak Na^+^ current density	Reduced	Reduced	Greater	N/A	N/A	Reduced	3, 9, 14
Na^+^ channel activation	No change	No change	No Change	N/A	N/A	Positive shift	15–19
						Negative shift	20
						No Change	21
Na^+^ channel inactivation	Negative shift	No change	No change	N/A	N/A	Positive shift	17, 21, 22
						Negative shift	15, 16, 18, 20
						No Change	18, 20
Na^+^ channel recovery from inactivation	No change	*Not assessed*	No Change	N/A	N/A	Faster	17–20, 22
						Slower	21
						No change	15, 16, 20

Key: 1 = Morita et al., 2003, 2 = Stokoe et al., 2007a, 3 = Head et al., 2005, 4 = Killeen et al., 2007, 5 = Balasubramaniam et al., 2003, 6 = Saumarez et al., 2003, 7 = Stokoe et al., 2007b, 8 = Watanabe et al., 2005, 9 = Papadatos et al., 2002, 10 = Sabir et al., 2007, 11 = Antzelevitch, 2001, 12 = Kurita et al., 2002, 13 = Thomas et al., 2007, 14 = Chen et al., 1998, 15 = Baroudi and Chahine, 2000, 16 = Wan et al., 2001, 17 = Zhang et al., 2007, 18 = Akai et al., 2000, 19 = Dumaine et al., 1999, 20 = Rook et al., 1999, 21 = Chen et al., 1998, 22 = Wang et al., 2000, 23 = Wilde et al., 2002. (“−” = absent; “+” = present).
